# Molecular mechanisms underlying bacterial resistance to ceftazidime/avibactam

**DOI:** 10.1002/wsbm.1571

**Published:** 2022-07-26

**Authors:** Luying Xiong, Xueting Wang, Yuan Wang, Wei Yu, Yanzi Zhou, Xiaohui Chi, Tingting Xiao, Yonghong Xiao

**Affiliations:** ^1^ Collaborative Innovation Center for Diagnosis and Treatment of Infectious Diseases, State Key Laboratory for Diagnosis and Treatment of Infectious Diseases, the First Affiliated Hospital, College of Medicine Zhejiang University Hangzhou China; ^2^ Jinan Microecological Biomedicine Shandong Laboratory Jinan China

**Keywords:** ceftazidime/avibactam, efflux pump, outer membrane protein, resistance mechanism, β‐lactamase

## Abstract

Ceftazidime/avibactam (CAZ/AVI), a combination of ceftazidime and a novel β‐lactamase inhibitor (avibactam) that has been approved by the U.S. Food and Drug Administration, the European Union, and the National Regulatory Administration in China. CAZ/AVI is used mainly to treat complicated urinary tract infections and complicated intra‐abdominal infections in adults, as well as to treat patients infected with Carbapenem‐resistant *Enterobacteriaceae* (CRE) susceptible to CAZ/AVI. However, increased clinical application of CAZ/AVI has resulted in the development of resistant strains. Mechanisms of resistance in most of these strains have been attributed to *bla*_KPC_ mutations, which lead to amino acid substitutions in β‐lactamase and changes in gene expression. Resistance to CAZ/AVI is also associated with reduced expression and loss of outer membrane proteins or overexpression of efflux pumps. In this review, the prevalence of CAZ/AVI‐resistance bacteria, resistance mechanisms, and selection of detection methods of CAZ/AVI are demonstrated, aiming to provide scientific evidence for the clinical prevention and treatment of CAZ/AVI resistant strains, and provide guidance for the development of new drugs.

This article is categorized under:

Infectious Diseases > Molecular and Cellular Physiology

## Graphical abstract

The molecular mechanisms to ceftazidime/avibactam resistance.

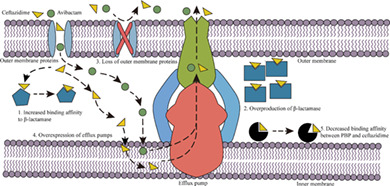

## 1 INTRODUCTION

Infections of patients with multidrug‐resistant Gram‐negative bacteria (MDRGNB) have become increasingly common. These bacteria have been associated with high morbidity and mortality rates, posing a serious threat to public health worldwide (Orsi et al., 2011). Carbapenems are considered one of the most powerful classes of therapeutic drugs for the treatment of MDRGNB infections; however, their use is limited by the emergence of carbapenemase‐producing bacteria (Daikos & Markogiannakis, 2011). The main carbapenemases produced by bacteria include *Klebsiella pneumoniae* carbapenemase (KPC), metallo‐β‐lactamase (MBL), and oxacillinase (OXA; Guh et al., 2015; Tamma et al., 2017). Carbapenemase‐resistant bacterial infections, however, may be susceptible to combination of β‐lactam and β‐lactamase inhibitors (BLI) with carbapenemase‐inhibiting activity (Bebrone et al., 2010).

**FIGURE 1:**
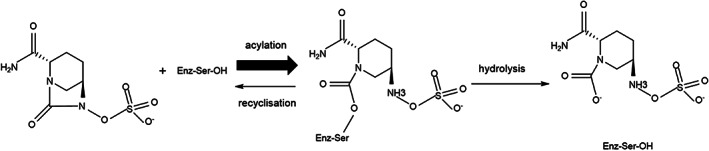
Schematic representation of the acylation, recyclization, and hydrolysis reactions between a serine β‐lactamase and avibactam

Common classical BLIs in clinical use include clavulanic acid, sulbactam, and tazobactam, all of which have β‐lactam structures. BLIs bind stably and irreversibly to β‐lactamases, initially forming an intermediate (E‐I), which undergoes an additional reaction to form free enzyme (E) and hydrolyzed clavulanic acid (I*), which is known as a “suicide enzyme inhibitor”. These classical BLIs are active against many class A β‐lactamases but do not interact with class B, C, and D β‐lactamases. With the emergence of other carbapenemases, some new non‐β‐lactam inhibitors have been recently approved for clinical use (Drawz et al., 2014). Relebactam is combined with imipenem to treat infections caused by CRE and *Pseudomonas aeruginosa*, including strains that produce KPC and class C β‐lactamases (Hirsch et al., 2012; Lapuebla et al., 2015; Livermore et al., 2013). Nacubactam, a diazabicyclooctane (DBO) inhibitor combined with meropenem for development, inhibits class A and C serine β‐lactamases (Morinaka et al., 2015). Zidebactam is another DBO inhibitor, combined with cefepime against *enterobacteriaceae* species and *P*. *aeruginosa* that express ESBLs, AmpC, KPC, and OXA‐48 β‐lactamases (D. M. Livermore, Mushtaq, et al., 2017). Vaborbactam, the first boronic acid BLI, is designed specifically to inhibit KPC carbapenemases. And it also has activity against other class A and class C β‐lactamases (Hecker et al., 2015). Meropenem–vaborbactam has shown good in vitro activity against *Enterobacter* spp., *Klebsiella* spp., and *Citrobacter* spp. that produce KPC enzymes (Castanheira et al., 2016; Lomovskaya et al., 2017). Avibactam (AVI) is a novel bridging DBO non‐β‐lactam BLI with weak antibacterial activity (Bonnefoy et al., 2004; Ehmann et al., 2012). However, AVI binds covalently and reversibly to A, C, and some D class serine β‐lactamases to form covalent compounds that inhibit the activity of these enzymes. The active enzyme and AVI can later be regenerated by reversible deacylation and recycling (Bush & Bradford, 2019). However, because the rate of AVI ring opening is much greater than the rate of cyclization, resulting in the maintenance of β‐lactamase inhibition, AVI has a long‐lasting enzyme inhibitory effect (Figure 1; Tooke et al., 2019). Unlike classical BLIs, AVI‐enzyme inhibitor complexes do not generally undergo hydrolysis; AVI undergoes a reaction with KPC‐2 similar to those of clavulanic acid and tazobactam with KPC‐2, with the complexes formed during the reaction between AVI and KPC‐2 able to undergo hydrolysis (Tooke et al., 2019).

**FIGURE 2:**
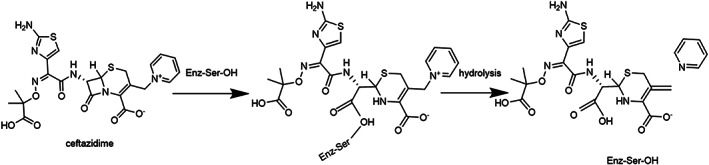
Example of an irreversible reaction between a β‐lactam and a nucleophilic serine enzyme. The figure shows the reaction of a penicillin with a penicillin binding protein (transpeptidase) to yield a stable acyl‐enzyme complex, which subsequently undergoes slow hydrolysis.

Ceftazidime/Avibactam (CAZ/AVI) is the first drug approved by the U.S. Food and Drug Administration for the treatment of CRE. Although CAZ is easily hydrolyzed by β‐lactamase when used alone (Figure 2), this combination, which is administered intravenously in a 4:1 ratio by weight (2.0 g CAZ/0.5 g AVI), has good antibacterial activity against carbapenemase‐producing Gram‐negative bacteria (Shirley, 2018). In October 2015, a KPC‐3‐producing strain of *K*. *pneumoniae* isolated from a blood culture of a 62‐year‐old woman not treated with CAZ/AVI was found to be resistant to CAZ/AVI by antimicrobial susceptibility testing, making this the first reported case of CAZ/AVI resistance (Humphries et al., 2015). Since then, many reports have described bacterial strains resistant to CAZ/AVI (Giddins et al., 2018; Winkler, Papp‐Wallace, & Bonomo, 2015). This review summarized the prevalence, resistant mechanism, and detection methods of CAZ/AVI‐resistant *Enterobacteriaceae*, *P*. *aeruginosa*, and *A*. *baumannii*, especially the influence of β‐lactamase variation on MIC values of CAZ/AVI, aiming at establishing the scientific and reasonable diagnosis and treatment plan, according to corresponding molecular mechanisms. It provides a theoretical basis for the rational use of CAZ/AVI in the treatment of bacterial infectious diseases in clinical practice.

## 2 PREVALENCE OF CAZ/AVI‐RESISTANT BACTERIA

### 2.1 Global resistance surveillance

According to the 2012–2016 ATLAS (Antimicrobial Testing Leadership and Surveillance), Global Resistance Surveillance reported that the overall resistance rate of Gram‐negative bacteria to CAZ/AVI was low (0.2%–8.1%). The resistance rate of *Enterobacteriaceae* to CAZ/AVI was less than 2.2%, with 0.2% for *Escherichia coli*, 1.2% for *K*. *pneumoniae*, and 2.2% for *Enterobacter cloacae*. While among CRE, the resistance rate of Carbapenem‐resistant *K*. *pneumoniae* (CRKP), Carbapenem‐resistant *E*. *coli* (CREC) and Carbapenem‐resistant *E*. *cloacae* were 14.4%, 27.7%, and 57.7%, respectively. The overall resistance rate of *P*. *aeruginosa* to CAZ/AVI was significantly higher than that of *Enterobacteriaceae* at 8.1%; Carbapenem‐resistant *P*. *aeruginosa* (CRPA) showed 25.5% resistance to CAZ/AVI. In addition, the resistance rate of CAZ/AVI varied greatly among *Enterobacteriaceae* and *P*. *aeruginosa* producing different carbapenemases: among *Enterobacteriaceae*, KPC‐producing strains were resistant to CAZ/AVI at 1.5%, and NDM‐producing strains at 99.6%, while among *P*. *aeruginosa*, KPC‐producing strains were resistant to CAZ/AVI at 35.7% and NDM‐producing strains at 97% (Table 1; Kiratisin et al., 2021; H. Zhang, Xu, et al., 2020).

**TABLE 1:** Resistance rate of pathogens against CAZ/AVI

Project	Region/country	Pathogen	R%	Charecteristics of pathogen	MIC50 (mg/L)	MIC90 (mg/L)	MIC range (mg/L)	Reference
INFORM (2012–2015)	Asia‐Pacific countries	*Enterobacteriaceae*	1.0		0.12	0.5	≤0.015 to >128	(Karlowsky et al., 2018)
		*Enterobacteriaceae*	98.6	MBL positive	>128	>128	2 to >128	
		*Enterobacteriaceae*	0.2	MBL negative	0.12	0.5	≤0.015 to >128	
		*E*. *coli*	0.1		0.12	0.25	≤0.015 to >128	
		*E*. *coli*	0.1	MBL negative	0.12	0.25	≤0.015 to >128	
		*K*. *pneumoniae*	1.7		0.12	0.5	≤0.015 to >128	
		*K*. *pneumoniae*	0.3	MBL negative	0.12	0.5	≤0.015 to >128	
		*K*. *oxytoca*	1.2		0.12	0.25	≤0.015 to >128	
		*K*. *oxytoca*	0.0	MBL negative	0.12	0.25	≤0.015 to 16	
		*Enterobacter* spp	1.9		0.25	1	≤0.015 to >128	
		*Enterobacter* spp	0.3	MBL negative	0.25	1	≤0.015 to 64	
		*P*. *aeruginosa*	7.4		2	8	0.03 to >128	
		*P*. *aeruginosa*	3.9	MBL negative	2	8	0.03 to >128	
INFORM (2012–2015)	Latin American	*Enterobacteriaceae*	0.3		0.12	0.5	≤0.015 to >128	(Karlowsky et al., 2019)
		*Enterobacteriaceae*	0.1	MBL negative	0.12	0.5	≤0.015 to >128	
		*Enterobacteriaceae*	94.1	MBL positive	64	>128	0.12 to >128	
		*E*. *coli*	0.1		0.12	0.25	≤0.015 to 32	
		*E*. *coli*	0.0	MBL negative	0.12	0.25	≤0.015 to 8	
		*K*. *pneumoniae*	0.5		0.25	1	≤0.015 to >128	
		*K*. *pneumoniae*	0.0	MBL negative	0.12	1	≤0.015 to >128	
		*P*. *aeruginosa*	12.6		2	16	0.12 to >128	
		*P*. *aeruginosa*	7.2	MBL negative	2	8	0.12 to >128	
		*P*. *aeruginosa*	94.5	MBL positive	32	>128	2 to >128	
INFORM (2012–2015)	European countries	Enterobacterales	0.6		0.12	0.5		(K. M. Kazmierczak, de Jonge, et al., 2018a)
		Enterobacterales	90.8	MBL‐positive	>128	>128	1 to >128	
		*P*. *aeruginosa*	7.6		2	8	0.03 to >128	(K. M. Kazmierczak, de Jonge, et al., 2018b)
		*P*. *aeruginosa*	97.2	MBL‐positive	32	>128	2 to >128	
ATLAS (2012–2016)	All regions	*E*. *coli*	0.2		0.12	0.25	0.015–256	(H. Zhang, Xu, et al., 2020)
		*E*. *coli*	27.7	CR	0.5	256	0.03–256	
		*K*. *pneumoniae*	1.2		0.12	1	0.015–256	
		*K*. *pneumoniae*	14.4	CR	1	256	0.015–256	
		*E*. *cloacae*	2.2		0.25	1	0.015–256	
		*E*. *cloacae*	57.7	CR	128	256	0.06–256	
		*P*. *aeruginosa*	8.1		2	8	0.015–256	
		*P*. *aeruginosa*	25.5	CR	4	64	0.015–256	
		*A*. *baumannii*			32	128	0.03–256	
		*A*. *baumannii*		CR	64	256	0.06–256	
INFORM (2015–2017)	Asia‐Pacific region	Enterobacterales	1.9		0.12	0.5	≤ 0.015 to ≥ 256	(Ko & Stone, 2020)
		Enterobacterales	0.0	MBL negative	1	2	0.12–4	
		Enterobacterales	100.0	MBL positive	≥ 256	≥ 256	32 to ≥ 256	
		*E*. *coli*	0.6		0.12	0.25	≤ 0.015 to ≥ 256	
		*E*. *coli*	100.0	CR and MBL positive	≥ 256	≥ 256	64 to ≥ 256	
		*K*. *pneumoniae*	2.5		0.12	0.5	≤ 0.015 to ≥ 256	
		*K*. *pneumoniae*	100.0	CR and MBL positive	≥ 256	≥ 256	32 to ≥ 256	
		*P*. *aeruginosa*	7.3		2	8	≤ 0.015 to ≥ 256	
		*P*. *aeruginosa*	31.4	MDR	8	128	0.25 to ≥ 256	
		*P*. *aeruginosa*	96.1	CR and MBL positive	128	≥ 256	2 to ≥ 256	
	European countries	*P*. *aeruginosa*	7.7		2	8	≤ 0.015 to ≥ 256	(Stone et al., 2020)
		*P*. *aeruginosa*	28.3	MDR	8	64	0.12 to ≥256	
		*P*. *aeruginosa*	82.7	CR	32	≥ 256	1 to ≥ 256	
		*P*. *aeruginosa*	95.3	MBL‐positive	64	≥ 256	1 to ≥ 256	
ATLAS (2016–2018)	Africa‐Middle East	Enterobacterales	39.5	CR		≥ 256	0.12 to ≥256	(Kiratisin et al., 2021)
		Enterobacterales	98.4	CR and MBL positive		≥ 256	8 to ≥256	
		*P*. *aeruginosa*	92.3	CR		128	2 to ≥256	
		*P*. *aeruginosa*	100.0	CR and MBL positive		128	16 to ≥256	
	Asia/South Pacific	Enterobacterales	66.6	CR		≥ 256	0.12 to ≥256	(Kiratisin et al., 2021)
		Enterobacterales	99.5	CR and MBL positive		≥ 256	1 to ≥256	
		*P*. *aeruginosa*	90.6	CR		≥ 256	2 to ≥256	
		*P*. *aeruginosa*	95.6	CR and MBL positive		≥ 256	2 to ≥256	
	Europe	Enterobacterales	28.0	CR		≥ 256	≤ 0.015 to ≥ 256	(Kiratisin et al., 2021)
		Enterobacterales	96.9	CR and MBL positive		≥ 256	1 to ≥ 256	
		*P*. *aeruginosa*	90.6	CR		≥ 256	1 to ≥256	
		*P*. *aeruginosa*	95.6	CR and MBL positive		≥ 256	1 to ≥256	
	Latin American	Enterobacterales	18.5	CR		≥ 256	≤ 0.015 to ≥ 256	(Kiratisin et al., 2021)
		Enterobacterales	97.4	CR and MBL positive		≥ 256	0.25 to ≥256	
		*P*. *aeruginosa*	82.9	CR		≥ 256	1 to ≥256	
		*P*. *aeruginosa*	96.0	CR and MBL positive		≥ 256	4 to ≥256	
	All regions	*P*. *aeruginosa*	35.7	KPC‐positive		64	1 to ≥256	(Kiratisin et al., 2021)
		*P*. *aeruginosa*	97.0	NDM‐positive		≥ 256	2 to ≥256	
		*P*. *aeruginosa*	37.0	GES‐positive		64	1 to ≥256	
		Enterobacterales	1.5	KPC‐positive		4	≤0.015 to ≥256	
		Enterobacterales	99.6	NDM‐positive		≥ 256	0.25 to ≥256	
INFORM (2015–2017)	All regions	Enterobacterales	27.0	Meropenem‐NS		≥ 256		(Spiliopoulou et al., 2020)
	Europe	Enterobacterales	23.2	Meropenem‐NS		≥ 256		
	Latin American	Enterobacterales	12.5	Meropenem‐NS		64		
	Asia	Enterobacterales	51.7	Meropenem‐NS		≥ 256		
	Africa/Middle East	Enterobacterales	49.2	Meropenem‐NS		≥ 256		
	Oceania	Enterobacterales	88.9	Meropenem‐NS		≥ 256		
INFORM (2015–2017)	Global	*K*. *pneumoniae*	4.7	MDR		2	≤0.015 to ≥256	(Rossolini & Stone, 2020)
		*K*. *pneumoniae*	0.1	MDR, ESBL‐positive, carbapenemase‐negative		1	≤0.015 to ≥256	
		*K*. *pneumoniae*	0.2	MDR, CR, MBL‐negative		2	≤0.015 to ≥16	
		*K*. *pneumoniae*	98.0	MDR, CR, MBL‐positive		≥ 256	2 to ≥256	
ATLAS (2015–2019)	Asia‐Pacific Region	*P*. *aeruginosa*	8.5		2	8	0.015–256	(Lee et al., 2022)
		*P*. *aeruginosa*	40.3	CR	8	256	0.03–256	
		*P*. *aeruginosa*	39.1	MDR	8	256	0.25–256	
		*P*. *aeruginosa*	94.0	MBL‐positive	256	256		
ATLAS (2017–2019)	Latin American	Enterobacterales	1.9		0.12	0.5		(Karlowsky et al., 2021)
		Enterobacterales	25.3	Meropenem‐NS	1	>128		
		Enterobacterales	8.2	Multidrug‐resisstant	0.5	4		
		Enterobacterales	0.5	KPC‐positive	0.5	2		
		Enterobacterales	98.6	MBL‐positive	>128	>128		
		*P*. *aeruginosa*	13.1		2	32		
		*P*. *aeruginosa*	38.1	Meropenem‐NS	8	64		
		*P*. *aeruginosa*	51.0	Multidrug‐resisstant	16	128		
		*P*. *aeruginosa*	35.3	KPC‐positive	8	32		
		*P*. *aeruginosa*	95.8	MBL‐positive	32	>128		

Abbreviations: ATLAS, Antimicrobial Testing Leadership and Surveillance; CR, carbapenem‐resistant; INFORM, the International Network for Optimal Resistance Monitoring; KPC, Klebsiella pneumonia carbapenemase; MBL, metallo‐β‐lactamase; MDR, multidrug‐resistant.

### 2.2 Regional resistance surveillance of *Enterobacteriaceae*

The 2012–2015 INFORM (the International Network for Optimal Resistance Monitoring, INFORM) resistance monitoring system showed that the overall resistance rate of *Enterobacteriaceae* to CAZ/AVI was low in different regions of the world, with resistance rates in Latin America and Europe is only 0.3%, while the resistance rate in the Asia Pacific is relatively higher, at 1.0% (Karlowsky et al., 2018, 2019; K. M. Kazmierczak, de Jonge, et al., 2018a). By 2015–2017, the resistance rate of *Enterobacteriaceae* to CAZ/AVI in the Asia‐Pacific region increased to 1.9%, and further investigation of the resistance rate of carbapenem nonsusceptible *Enterobacteriaceae* to CAZ/AVI in different regions showed that Oceania had the highest CAZ/AVI resistance rate of 88.9%, followed by Asia (51.7%), Africa‐Middle East (49.2%), Europe (23.2%), and the lowest resistance rate was found in Latin America (12.5%; Spiliopoulou et al., 2020). By 2017–2019, carbapenem nonsusceptible *Enterobacteriaceae* bacteria in Latin America increased their resistance rate to CAZ/AVI nearly 1‐fold to 25.3% (Karlowsky et al., 2021). In addition, global resistance surveillance by the ATLAS project from 2012 to 2016 showed that carbapenem‐resistant *Enterobacteriaceae* had a resistance rate of 57.7% to CAZ/AVI, followed by CREC with a resistance rate of 27.7% to CAZ/AVI (H. Zhang, Xu, et al., 2020). The distribution of resistance rate of CRE to CAZ/AVI in different regions was as follows: the highest resistance rate to CAZ/AVI was 66.6% in the Asia‐Pacific region, followed by Africa‐Middle East (39.5%), Europe (28.0%), and Latin America (18.5%), whose trend was consistent with the 2015–2017 INFORM surveillance system showing the distribution of resistance rates of carbapenem nonsusceptible *Enterobacteriaceae* to CAZ/AVI (Table 1). These results suggest that CRE resistance to CAZ/AVI varies by region and that the differences in CAZ/AVI resistance rates are likely due to the different percentages of MBLs in different regions (Kiratisin et al., 2021; Spiliopoulou et al., 2020).

### 2.3 Regional resistance surveillance of *P*. *aeruginosa*

*Pseudomonas aeruginosa* has a higher resistance rate to CAZ/AVI compared to *Enterobacteriaceae*. INFORM resistance surveillance system in 2012–2015 showed the highest resistance rate to CAZ/AVI in Latin America at 12.6%, followed by Europe (7.6%) and the Asia Pacific (7.4%; Karlowsky et al., 2018, 2019; K. M. Kazmierczak, de Jonge, et al., 2018b). In recent years, the resistance rate of P. aeruginosa to CAZ/AVI has been relatively stable across continents, with 13.1% in Latin America, 7.7% in Europe, and 7.3% in the Asia Pacific (Karlowsky et al., 2021; K. M. Kazmierczak, de Jonge, et al., 2018b; Ko & Stone, 2020; Lee et al., 2022). Among carbapenemase‐producing *P*. *aeruginosa*, the resistance rate to CAZ/AVI was high in all continents: 92.3% in Africa‐Middle East, 90.6% in the Asia Pacific and Europe, and 82.9% in Latin America (Kiratisin et al., 2021), showing that carbapenemase production correlates strongly with CAZ/AVI resistance (Table 1).

### 2.4 Resistance surveillance in China

Evaluation of 10,661 Gram‐negative strains collected by the Blood Bacterial Resistant Investigation Collaborative System in China from January 2018 to December 2019 showed that 1% of *E*. *coli* strains were resistant to CAZ/AVI (MIC50, 0.25/4 mg/L; MIC90, 1/4 mg/L), including 60.7% of CREC strains. In addition, 1.6% of *K*. *pneumoniae* strains were resistant to CAZ/AVI (MIC50, 0.5/4 mg/L; MIC90, 4/4 mg/L), including 8.3% of CRKP strains (MIC50, 4/4 mg/L; MIC90, 8/4 mg/L). Also, 2.8% of *P*. *aeruginosa* strains were resistant to CAZ/AVI (MIC50, 4/4 mg/L; MIC90, 8/4 mg/L), including 11.6% of CRPA strains (MIC50, 4/4 mg/L. MIC90, 16/4 mg/L; T. Xu, Guo, et al., 2021). Furthermore, data from the China Antimicrobial Surveillance Network (CHINET) show that in 2017, the CAZ/AVI resistance rates of *Enterobacteriaceae* and *P*. *aeruginosa* were 5.4% (MIC50, ≤0.25/4 mg/L; MIC90, 2/4 mg/L) and 13.5% (MIC50, 2/4 mg/L; MIC90, 16/4 mg/L), respectively. The CAZ/AVI resistance rate was higher for CRE, at 24.7% (MIC50, 2/4 mg/L; MIC90, >32/4 mg/L), among which the resistance rate of CREC to CAZ/AVI was up to 71.4% (Yin et al., 2019). Resistance rates to CAZ/AVI were slightly higher in 2018, up to 6.0% (MIC50, 0.25/4 mg/L; MIC90, 2/4 mg/L) for *Enterobacteriaceae*, 35.7% (MIC50, 8/4 mg/L; MIC90, 64/4 mg/L) for CRPA and 90.9% for CREC (MIC50, >64/4 mg/L. MIC90, >64/4 mg/L; Yang et al., 2020).

## 3 MECHANISMS UNDERLYING RESISTANCE TO CAZ/AVI

Most MBLs have the metal ion Zn^2+^ at their active site; whereas other β‐lactamases have a serine (Ser70) at their active site; the latter are called serine β‐lactamases. The most common groups of MBLs are the Verona integrin‐encoded MBLs (VIMs), imipenemases (IMPs), and New Delhi MBLs (NDMs). MBL‐producing bacteria are naturally resistant to CAZ/AVI because their MBLs do not contain a serine at their active site (Centers for Disease Control and Prevention (CDC), 0). Evaluation of *Enterobacteriaceae* and *P*. *aeruginosa* collected from 42 medical centers in nine countries in the Asia‐Pacific region by the INFORM during the years 2012–2015 showed that 1% (91/9149) and 7.4% (151/2038), respectively, were resistant to CAZ/AVI, with MBL‐positive strains accounting for 80.2% (73/91) and 48.3% (73/151), respectively, of these CAZ/AVI‐resistant strains (Karlowsky et al., 2018). Of the 372 CRE strains collected by CHINET from more than 30 medical centers in 2017, 92 (24.7%) were resistant to CAZ/AVI, with 66 (71.7%) of these containing *bla*_NDM_. Of the 134 CAZ/AVI‐resistant strains collected in 2018, 57 (42.5%) contained *bla*_NDM_, whereas of the 30 CAZ/AVI‐resistant CREC, 21 (70%) were positive for NDM (Yang et al., 2020; Yin et al., 2019). Taken together, these findings indicate that MBL‐producing strains account for a high proportion of CAZ/AVI‐resistant strains.

By contrast, the main mechanisms underlying resistance to CAZ/AVI in Gram‐negative bacteria that do not produce MBL include: (1) β‐lactamase variants; (2) changes in bacterial membrane permeability; (3) increased expression of efflux pumps; and (4) mutation of PBPs (Shi et al., 2020).

### 3.1 β‐Lactamase variants

β‐Lactamase variants are an important mechanism of bacterial resistance to CAZ/AVI. Alteration of key residues at the active sites of serine β‐lactamases can increase their MIC values for CAZ/AVI significantly (Table 2). Serine β‐lactamases are dependent on several highly conserved sequences responsible for recognition of, and reactions with, antibiotics. These sequences include the Ω‐loop, consisting of amino acids 164–179. Glu166 and Asn170 are involved in the acylation and deacylation of antibiotics by β‐lactamases. The Ω‐loop acts as a structural domain unit that interacts with antibiotics, forming the binding cavity for β‐lactamase‐substrate action. This allows the carbonyl on the β‐lactam ring of the antibiotic to polarize, forming an acylated enzyme complex. The amino acid substitution in the KPC enzyme alters the structure of the binding cavity, thereby reducing resistance to the binding of CAZ and the active site of the enzyme, thereby increasing β‐lactamase binding and hydrolysis of CAZ, an important mechanism of bacterial resistance to CAZ/AVI. Individual amino acid substitutions, particularly at positions 164, 167, 169, and 179 of the Ω‐loop, increase the affinity of the β‐lactamase to CAZ and increase its hydrolysis (Winkler, Papp‐Wallace, & Bonomo, 2015). In addition, amino acid substitutions in the Ω‐loop can reduce the binding of β‐lactamase to AVI, weakening the inhibitory effect of AVI on β‐lactamase and increasing hydrolysis of CAZ (F. Compain et al., 2020a, 2020b). Alterations in β‐lactamase expression can also lead to CAZ/AVI resistance (Humphries et al., 2015).

**TABLE 2:** CAZ/AVI resistance caused by β‐lactamase mutations

β‐Lactamase	Variation	CAZ/AVI MIC	Possible mechanisms	Year/pathogen	Country	Notes	References
KPC‐3	Asp179Tyr	>256/4	Increased affinity for CAZ and restricted AVI binding	2018/kpn	United States	Clinical strain	(Giddins et al., 2018)
	Ala172Thr	12/4	Increased affinity for CAZ	2021/kpn	France	Clinical strain	(Jousset et al., 2021)
	Asp179Tyr/Ala177Glu	128/4 or 256/4	Increased affinity for CAZ and restricted AVI binding	2017/kpn	United States	Clinical strain	(Shields, Chen, et al., 2017)
	Asp179Tyr/Thr243Met	>256/4	Increased affinity for CAZ and restricted AVI binding	2017/kpn	United States	Clinical strain	(Shields, Chen, et al., 2017)
	Val240Gly	32/4	Increased affinity for CAZ and restricted AVI binding	2017/kpn	United States	Clinical strain	(Shields, Chen, et al., 2017)
	167–168Glu‐Leu del	16/4	Increased affinity for CAZ and restricted AVI binding	2020/kpn	Italy	Clinical strain	(Antinori et al., 2020)
	276–277 Glu‐Ala‐Val ins	>256/4	Increased affinity for CAZ and restricted AVI binding	2019/kpn	Switzerland	Clinical strain	(Poirel et al., 2020)
	269–270 Pro‐Asn‐Lys ins	>128/4	Increased affinity for CAZ and restricted AVI binding	2019/kpn	Switzerland	Clinical strain	(Mueller et al., 2019)
	Val240Ala	16/4	Increased affinity for CAZ and restricted AVI binding	2019/kpn	Greece	Clinical strain	(Galani et al., 2019)
	Ser181 ins + Thr244Ala	>256/4	Increased affinity for CAZ and restricted AVI binding	2020/kpn	Rome	Clinical strain	(Venditti et al., 2021)
	Asp163Gly	32/4	Increased affinity for CAZ and restricted AVI binding	2015/kpn	United Kingdom	Laboratory selection strain	(Livermore et al., 2015)
	180–181 Ser ins	64/4	Increased affinity for CAZ and restricted AVI binding	2015/kpn	United Kingdom	Laboratory selection strain	(Livermore et al., 2015)
	181–182 Ser‐Ser ins	32/4	Increased affinity for CAZ and restricted AVI binding	2015/ecl	United Kingdom	Laboratory selection strain	(Livermore et al., 2015)
	Thr243Pro	32/4	Increased affinity for CAZ and restricted AVI binding	2015/ecl	United Kingdom	Laboratory selection strain	(Livermore et al., 2015)
	265–266 Ala‐Arg ins	16/4	Increased affinity for CAZ and restricted AVI binding	2015/ecl	United Kingdom	Laboratory selection strain	(Livermore et al., 2015)
	183–184 Arg‐Ala‐Val‐Thr‐Thr‐Ser‐Ser‐Pro ins	128/4	Increased affinity for CAZ and restricted AVI binding	2015/ecl	United Kingdom	Laboratory selection strain	(Livermore et al., 2015)
KPC‐2	Asp179Asn	64/4	Increased affinity for CAZ and reduced inhibitory capacity of avibactam	2015/eco	United States	Laboratory selection strain	(Winkler, Papp‐Wallace, & Bonomo, 2015)
	165–166 Gln‐Leu ins	16/4	Reduced inhibitory capacity of avibactam	2021/kpn	China	Laboratory selection strain	(Guo et al., 2021)
	Leu169Pro plus Ser181ins	128/4	Reduced inhibitory capacity of avibactam	2021/kpn	China	Laboratory selection strain	(Guo et al., 2021)
	166–167 Gln‐Leu del	32/4	Reduced inhibitory capacity of avibactam	2021/kpn	China	Laboratory selection strain	(Guo et al., 2021)
	Asp179Tyr	32/4	Increased affinity for CAZ and reduced inhibitory capacity of avibactam	2020/kpn	China	Clinical strain	(P. Zhang, Shi, et al., 2020)
	Arg164Ala	16/4	Increased affinity for CAZ and restricted AVI binding	2015/eco	United States	Laboratory selection strain	(Winkler, Papp‐Wallace, & Bonomo, 2015)
	Arg164Pro	64/4	Increased affinity for CAZ and restricted AVI binding	2015/eco	United States	Laboratory selection strain	(Winkler, Papp‐Wallace, & Bonomo, 2015)
	Asp179Ala	64/4	Increased affinity for CAZ and restricted AVI binding	2015/eco	United States	Laboratory selection strain	(Winkler, Papp‐Wallace, & Bonomo, 2015)
	Asp179Gln	32/4	Increased affinity for CAZ and restricted AVI binding	2015/eco	United States	Laboratory selection strain	(Winkler, Papp‐Wallace, & Bonomo, 2015)
	242–243Gly‐Thr del	>16/4	Increased affinity for CAZ and restricted AVI binding	2003/kpn	United States	Clinical strain	(Niu et al., 2020)
CTX‐M‐15	Gln169Leu + Gly130Ser	16/4	Increased affinity for CAZ and reduced inhibitory capacity for avibactam	2018/kpn	France	Clinical strain	(Compain et al., 2018)
CTX‐M‐14	Pro170Ser + Thr264Ile	32/4	Increased hydrolytic activity toward CAZ	2017/kpn	Germany	Clinical strain	(Both et al., 2017)
AmpC	245–249 (Asp245, Ala246, Glu247, Gly248 and Tyr249) del	256/4	Increased hydrolytic activity toward CAZ and decreased AVI inhibition	2015/pae	United Kingdom	Laboratory selection strain	(Lahiri et al., 2015)
	238–244 (Arg238, Val239, Gly240, Pro241, Gly242, Pro243 and Leu244) del	64/4	Increased hydrolytic activity toward CAZ and reduced inhibitory capacity of avibactam	2015/pae	United Kingdom	Laboratory selection strain	(Lahiri et al., 2015)
	Asn346Tyr	16/4	Loss of hydrogen interactions with AVI, reducing the inhibitory capacity of avibactam	2018/ecl	United Kingdom	laboratory selection strain	(F. Compain et al., 2020a, 2020b)
	Gly176Arg	64/4	Increased affinity with CAZ	2018/ecl	United Kingdom	Laboratory selection strain	(Livermore et al., 2018)
	Arg168Pro	16/4	Decreased affinity with AVI	2018/ecl	United Kingdom	Laboratory selection strain	(Livermore et al., 2018)
	Asn366Tyr	16/4	Decreased affinity with AVI	2018/ecl	United Kingdom	Laboratory selection strain	(Livermore et al., 2018)
	Arg168His	32/4	Increased affinity with CAZ	2018/ecl	United Kingdom	Laboratory selection strain	(Livermore et al., 2018)
	Gly176Asp	16/4	Increased affinity with CAZ	2018/ecl	United Kingdom	Laboratory selection strain	(Livermore et al., 2018)
	Gly183Asp	32/4	–	2017/ecl	United States	Clinical strain	(MacVane et al., 2017)
	290–292 Lys‐Val‐Ala del plus Asn346Ile	128/4	Increased hydrolytic activity toward CAZ and reduced inhibitory capacity of avibactam	2021/kpn	China	Clinical strain	(M. Xu, Guo, et al., 2021)
OXA‐2	Asp149 dup	>32/4	–	2017/pae	Spain	Clinical strain	(Fraile‐Ribot et al., 2017)
OXA‐23405866	–	≥64/4	Natural drug resistance	2015/aba	Japan	clinical strain	(Yoshizumi et al., 2015)1
VEB‐1	Lys234Arg	16/4	Decreased ability to bond with AVI	2019/kpn	Greece	Clinical strain	(Galani et al., 2020)

Abbreviations: del: deletion; dup: duplication; ecl: *Enterobacter cloacae*; eco: *Escherichia coli*; ins: insertion; kpn: *Klebsiella pneumoniae*; pae: *Pseudomonas aeruginosa*.

#### 3.1.1 Alterations in KPC enzymes

KPC‐2 and KPC‐3, which are distributed widely worldwide, are not only present in *K*. *pneumoniae*, but have also been detected in other *Enterobacteriaceae*, including *Acinetobacter baumannii* and *P*. *aeruginosa* (Bush & Bradford, 2019). The main mechanisms of resistance associated with KPC are mutations in the *bla*_KPC_ gene and increased *bla*_KPC_ expression.

On the one hand, amino acid substitution in KPC caused by *bla*_KPC_ mutation can lead to CAZ/AVI resistance. One of the most prevalent amino acid substitutions in KPC‐2 and KPC‐3 is Asp179Tyr (Giddins et al., 2018; Shields, Nguyen, et al., 2017b): this amino acid substitution, as well as others in KPC‐3, such as Val240Gly, Asp179Tyr/Ala177Glu, and Asp179Tyr/Thr243Met, leads to CAZ/AVI resistance (Gaibani et al., 2018; Galani et al., 2019; Giddins et al., 2018; Haidar et al., 2017; Shields et al., 2018; Shields, Nguyen, et al., 2017a). Moreover, amino acid substitutions at different sites are associated with varying degrees of increased MIC levels: Asp179Tyr/Thr243Met (MIC value 256/4 mg/L) ≥ Asp179Tyr/Ala177Glu (MIC value 128/4–256/4 mg/L) > Asp179Tyr (MIC value 128/4 mg/L) > Val240Gly (MIC value 32/4 mg/L; Haidar et al., 2017; Shields, Chen, et al., 2017; Shields, Nguyen, et al., 2017a).

Experimentally, mutations have been introduced into *bla*_KPC_ by site‐directed mutagenesis, with the plasmids containing mutant *bla*_KPC‐3_ used to transform *E*. *coli* DH 5α by heat shock. The MIC value of native *bla*_KPC_ toward CAZ/AVI is 0.5/4 mg/L, but is altered by mutation to 16/4 mg/L for Asp179Tyr/Thr243Met, 8/4 mg/L for Asp179Tyr and Thr243Met, and 4/4 mg/L for the 165–166 Glu‐Leu insertion (Haidar et al., 2017). The greater change in MIC value for the Asp179Tyr mutation than for the 165–166 Glu‐Leu insertion or the Thr243Met mutation in CAZ/AVI suggests that the Ω‐loop plays a central role in maintaining the stability of the KPC enzymes. The Ω‐loop is formed by a salt bridge between Asp at position 179 and Arg at position 164. The less stable Asp179Tyr mutant has increased affinity for CAZ, and restricted binding to AVI. The 167–168Glu‐Leu deletion in the Ω‐loop of KPC‐3 was also found to increase the MIC value of CAZ/AVI from 1/4 to 16/4 mg/L, resulting in a phenotypic shift from susceptible to resistant (Antinori et al., 2020).

Similarly, KPC‐2 mutants containing Arg164Ala, Arg164Pro, Asp179Ala, Asp179Gln, and Asp179Asn amino acid substitutions are all resistant to CAZ/AVI, with MIC values of 16/4, 64/4, 64/4, 32/4, and 64/4 mg/L, respectively. To further study the mechanism by which KPC mutants increase MIC values for CAZ/AVI, the β‐lactamase kinetics and the inhibitory activity of AVI were evaluated in the Arg164Ala and Asp179Asn mutants. Evaluation of their catalytic activities using nitrocefin kinetic assays showed that the Arg164Ala variant had a 10‐fold higher *K*_m_ for nitrofecin than wild‐type KPC‐2. Rapid‐mixing stopped‐flow kinetic assays of KPC‐2 and the Asp179Asn variant on a 1.5 ms timescale revealed that the Asp179Asn variant revealed a phenomenon called “burst”. This “burst” contributed to the resistance of the enzyme to CAZ, and its inability to bind to penicillin binding proteins (PBPs). Evaluation of the *K*_i app_ and *K*_2_/*K* values of the Arg164Ala and Asp179Asn variants showed that similar concentrations of AVI were needed to fully inhibit both. These findings suggested that increased affinity for CAZ may prevent the binding of AVI to enzymes with Ω‐loop substitutions, thereby increasing MIC values for CAZ/AVI rather than reducing AVI inhibition (Winkler, Papp‐Wallace, & Bonomo, 2015). The Asp179Tyr variant of KPC‐2, called KPC‐33, which is induced by selective pressure asserted by CAZ/AVI, showed an increase in the MIC value for CAZ/AVI from 0.125/4 to 16/4 mg/L, while at the same time restoring susceptibility to carbapenem antibiotics, which is useful for treatment of infection by CAZ/AVI‐resistant strains (P. Zhang, Shi, et al., 2020). A strain containing the Ala172Thr variant of KPC‐3, called KPC‐39, was found to have a MIC value for CAZ/AVI of 12/4 mg/L. A study of its molecular structure showed that the Ala172Thr substitution enlarged the spatial structure of the Ω‐loop active site, enabling it to better accommodate CAZ and increase the affinity between CAZ and the variant enzyme, resulting in a resistant phenotype (Jousset et al., 2021).

In addition to the Ω‐loop amino acid substitutions, some other substitutions can alter the affinity of the enzyme for CAZ, leading to CAZ/AVI resistance. The Asp163Gly substitution adjacent to the Ω‐loop in KPC‐3 increased the MIC of *K*. *pneumoniae* clinical isolates for CAZ/AVI from 1/4 to 32/4 mg/L; the 181_182Ser‐Ser insertion increased the MIC from 1/4 to 64/4 mg/L; the 180_181Ser insertion increased the MIC of *Enterobacter cloacae* for CAZ/AVI from 0.25/4 to 32/4 mg/L, whereas the 183_184Arg‐Ala‐Val‐Thr‐Thr‐Ser‐Ser‐Pro insertion increased the MIC from 0.5/4 to 128/4 mg/L (Livermore et al., 2015). A novel CAZ/AVI‐resistant mutant, KPC‐14, containing a two‐amino acid deletion in KPC‐2 (242_243Gly‐Thr) and isolated from patients in New York in 2020, has a MIC value for CAZ/AVI of >16/4 mg/L. This enzyme was significantly more efficient than KPC‐2 at catalyzing CAZ, but did not affect AVI inhibitory properties, suggesting that CAZ/AVI resistance may be due to an increase in β‐lactamase hydrolysis of CAZ rather than a decrease in its inhibition by AVI (Niu et al., 2020). In addition, KPC‐3 variants from clinical isolates of *K*. *pneumoniae* in Switzerland were resistant to CAZ/AVI (Galani et al., 2019; Gregory et al., 2010; Mueller et al., 2019; Poirel et al., 2020). These included KPC‐41, with a 269_270 Pro‐Asn‐Lys insertion and a MIC >128/4 mg/L; KPC‐50, with a 276_277Glu‐Ala‐Val insertion and a MIC of 256/4 mg/L; KPC‐8, with a Val240Gly substitution and a MIC of 32/4 mg/L; and KPC‐23, with a Val240Ala substitution and a MIC of 16/4 mg/L. In addition, combinations of these alterations, such as KPC‐64, a KPC‐3 variant with a Ser181 insertion, and Tyr244Ala substitution had a MIC >256/4 mg/L and were resistant to CAZ/AVI (Venditti et al., 2021).

Bacterial resistance to CAZ/AVI is due not only to the altered affinity of KPC variants for CAZ, but also to the altered ability of AVI to inhibit these enzymes. A mutant containing a KPC‐2 variant with a Asp179Tyr substitution (Barnes et al., 2017; Compain & Arthur, 2017; Hemarajata & Humphries, 2019) was found to have a CAZ/AVI MIC of 32/4 mg/L, whereas the CAZ/AVI MIC for wild‐type KPC‐2 was 1/4 mg/L. The Asp179Tyr substitution reduced the AVI rate constant for KPC‐2 inhibition by approximately 70,000‐fold, suggesting that the resistance of this variant to CAZ/AVI was also partly due to a significant reduction in the ability of AVI to inhibit the enzyme.

Culture of strains susceptible to CAZ/AVI in LB medium containing CAZ/AVI for 50 generations resulted in the generation of several variants of KPC‐2, such as a 165_166 Gln‐Leu insertion, a Leu169Pro insertion plus a Ser181 insertion, and a 166_167 Gln‐Leu deletion. Introduction of these *bla*_KPC_ mutations into a wild‐type strain susceptible to CAZ/AVI increased the MIC values for CAZ/AVI from 2/4, 4/4, and 0.25/4 mg/L to 16/4, 128/4, and 32/4 mg/L, respectively, but did not affect MIC values for CAZ significantly, suggesting that these variants mainly affect the inhibitory activity of AVI (Guo et al., 2021).

On other hand, increased expression of *bla*_KPC_ can also contribute to CAZ/AVI resistance. The first KPC‐3‐producing *K*. *pneumoniae* strain resistant to CAZ/AVI did not contain mutations in the *bla*_KPC‐3_ gene (Humphries et al., 2015). Real‐time quantitative PCR analysis, however, found that expression of the *bla*_KPC‐3_ gene was 3.8 ± 0.2‐fold higher in this resistant strain than in susceptible strains (Humphries & Hemarajata, 2017). Moreover, *bla*_KPC‐3_ gene expression was found to be 2.5‐fold or 1.5‐fold higher in resistant strains than in wild‐type strains (Gaibani et al., 2020). A study comparing 12 strains resistant to CAZ/AVI carrying wild‐type *bla*_KPC‐2_ with five strains susceptible to CAZ/AVI, also carrying *bla*_KPC‐2_, found that the number of copies of *bla*_KPC‐2_ was 2.5‐fold higher, and its expression 2.7‐fold higher, in resistant than in susceptible strains (P. Zhang, Shi, et al., 2020). Evaluation of two CAZ/AVI‐resistant strains isolated from a patient who had not been treated with CAZ/AVI showed that both strains carried two copies of the transposon *Tn4401a* encoding the KPC‐3 enzyme, thereby increasing the number of *bla*_KPC_ gene copies as well as its expression in the bacteria (Coppi et al., 2020). Moreover, increased expression of *bla*_KPC_ resulted in the incorporation of structural changes in the outer membrane proteins, thereby reducing outer membrane protein pore size and permeability, leading (ultimately) to reduced resistance to CAZ/AVI (Coppi et al., 2020). In addition, the increase in *bla*_KPC‐3_ expression was due to the transposition of the Tn4401 transposon carrying *bla*_KPC‐3_ onto the plasmid plncX3, thereby increasing the number of *bla*_KPC‐3_ copies (Nelson et al., 2017). Taken together, these findings show that increases in the number of copies and expression of *bla*_KPC_ are associated with CAZ/AVI resistance, and are most likely caused by an increase in the number of plasmids carrying *bla*_KPC_ or an increase in the number of copies of *bla*_KPC_ on the same plasmid.

#### 3.1.2 *bla*_CTX‐M_ and *bla*_SHV_ mutations lead to CAZ/AVI resistance

CTX‐M enzymes and SHV enzymes are not common in strains resistant to CAZ/AVI; however, they deserve attention. For example, a *K*. *pneumoniae* strain carrying wild‐type *bla*_CTX‐M‐14_ had a MIC value for CAZ/AVI of 1/4 mg/L, whereas a *K*. *pneumoniae* strain showing both high expression of *bla*_OXA‐48_ and a mutant *bla*_CTX‐M‐14 △170△264_ encoding a CTX‐M‐14 enzyme with Pro170Ser and Thr264Ile substitutions, showed an increase in the MIC value from 1/4 to 32/4 mg/L. To further confirm the role played by *bla*_CTX‐M‐14 △170△264_, its introduction into *E*. *coli* TOP 10 increased the MIC value for CAZ/AVI by 16‐fold, from 0.5/4 to 8/4 mg/L, and increased the MIC value for CAZ by 64‐fold, from 4/4 to 256/4 mg/L, suggesting that resistance of the mutant to CAZ/AVI is likely due to increased hydrolysis of CAZ. An Asp182Tyr substitution in the CTX‐M‐15 enzyme is also associated with decreased susceptibility to CAZ/AVI, with a 16‐fold increase in its MIC value for CAZ/AVI (Both et al., 2017; Dulyayangkul et al., 2020; Livermore et al., 2018). In addition, a variant of CTX‐M‐15 harboring two amino acid substitutions, Gln169Leu and Gly130Ser, induced resistance to CAZ/AVI in an *E*. *coli* strain susceptible to this combination (Compain et al., 2018). The Km value of WT CTX‐M‐15 was 10‐fold higher than that of the mutant enzyme, with the mechanism of resistance associated with increased affinity of the mutant enzyme for CAZ. CTX‐M‐15 includes several conserved sequences, such as 166_170 Glu‐Pro‐Thr‐Leu‐Asn on the 19‐residue Ω‐loop constituting the floor of active site. In addition, the conserved sequence 130_132 Ser‐Asp‐Asn is situated on a short loop in the all‐alpha domain, where it forms one side of the catalytic cavity (Compain et al., 2018). The structural features of CTX‐M‐15 suggest that the Pro170Ser substitution enhances the ability of the mutant enzyme to hydrolyze CAZ by altering the conformation of the Ω‐loop, thereby enabling CAZ to more easily enter the active site of the mutant enzyme. In addition, the Gly130Ser substitution may alter the structure of the catalytic cavity, enabling CAZ to more easily enter the catalytic cavity, as well as increasing the affinity between the variant enzyme and CAZ, leading ultimately to CAZ/AVI resistance.

The Ser130Gly substitution in the SHV‐1 enzyme was found to significantly reduce AVI‐induced carbamylation of β‐lactamase, with a 1700‐fold higher concentration of AVI required to inhibit the mutant than to inhibit wild‐type SHV‐1 β‐lactamase (Winkler, Papp‐Wallace, Taracila, & Bonomo, 2015).

#### 3.1.3 *ampC* mutations cause CAZ/AVI resistance

Among the class C β‐lactamases, AmpC enzyme variants are the most common. The wild‐type AmpC enzyme has a MIC value for CAZ/AVI of 4/4 mg/L, whereas deletion of amino acid residues from its Ω‐loop, such as the 245_249 Asp‐Ala‐Glu‐Gly‐Tyr and 238_244 Arg‐Val‐Gly‐Pro‐Gly‐Pro‐Leu deletions, increase the MIC values for CAZ/AVI from 4/4 mg/L to 256/4 and 64/4 mg/L, respectively (Lahiri et al., 2015).

The Asn346Tyr amino acid substitution in AmpC _cloacae_, DHA‐1, and PDC‐5 affects the ability of AVI to inhibit these enzymes. In the wild‐type enzyme, the carboxamide at Asn346 forms hydrogen bonds with avibactam sulfonate. The amino acid substitution at this position results in loss of this interaction, which is likely reinforced by steric hindrance due to the bulky Tyr side chain, resulting in a significant reduction in carbamoylation activity (Compain et al., 2020a, 2020b). In addition, the Gly183Asp amino acid substitution, caused by a nonsynonymous G548A nucleotide mutation in *bla*_PDC‐5_, results in resistance to CAZ/AVI, thereby increasing the MIC of *P*. *aeruginosa* against CAZ/AVI from 4/4 to ≥32/4 mg/L (MacVane et al., 2017). Moreover, AmpC enzyme variants containing Gly176Arg, Gly176Asp, Arg168Pro, Asn366Tyr, and Arg168His amino acid substitutions, generated in *Enterobacter cloacae* by stepwise induction, were resistant to CAZ/AVI, with MIC values ranging from 0.5/4 mg/L in wild‐type to 64/4, 16/4, 16/4, and 32/4 mg/L, respectively, in the mutants. However, the mechanisms of resistance associated with these amino acid substitutions differed. Specifically, the Arg168Pro substitution reduced the affinity of the AmpC enzyme for AVI, and eliminated the synergy between CAZ and AVI, whereas the Arg168His and Gly176Arg/Asp substitutions increased the affinity of the enzyme for CAZ (Lahiri et al., 2014). Because the Asn346 residue is key to AVI binding, the Asn346Tyr substitution reduces the ability of AVI binding, resulting in resistance to CAZ/AVI (Lahiri et al., 2014). Moreover, CAZ/AVI resistance in ESBL‐producing mutants is associated mostly with changes in the efflux pump, membrane permeability, or β‐lactamase expression (Livermore et al., 2018). Compared with CYM‐2, CYM‐172 has a 290_292 Lys‐Val‐Ala deletion and a Asn346Ile substitution. Introduction of this mutant gene into *E*. *coli* DH5α through plasmid conjugation increases the MIC value for CAZ/AVI to 16/4 or 32/4 mg/L. CAZ/AVI resistance may be due to a change in CMY structure caused by the deletion of amino acids in the R2‐loop of the enzyme, which may increase CAZ hydrolysis. Additionally, the Asn346Ile substitution results in steric hindrance between the Ile side chain and the sulfate group of AVI, thereby altering the binding affinity of the latter for the enzyme and resulting in CAZ/AVI resistance (M. Xu, Zhao, et al., 2021).

In addition to mutations and increased expression of the *ampC* gene, AVI induces AmpC enzyme activity. To test the ability of AVI to induce AmpC enzyme activity, strains of *E*. *cloacae*, *Citrobacter freundii*, and *P*. *aeruginosa* were incubated with 1, 4, and 32 mg/L AVI. At a concentration of 32 mg/L, AVI strongly induced expression of the AmpC enzyme in two of five *E*. *cloacae* and in two of five *P*. *aeruginosa* strains, with the enzyme content being thousands‐fold higher than prior to induction. These findings suggest that the affinity of AmpC variants for AVI is reduced, but that these enzymes can be induced by AVI, resulting in CAZ/AVI resistance (D. M. Livermore, Jamrozy, et al., 2017).

#### 3.1.4 *bla*_OXA_ mutations lead to CAZ/AVI resistance

The MICs of 265 OXA‐48‐producing strains (collected by the International Optimal Resistance Monitoring Network between 2012 and 2015) for CAZ/AVI ranged from 0.03 to >128 mg/L, with 7.5% of these strains being resistant to CAZ/AVI (K. M. Kazmierczak, Bradford, et al., 2018). The presence of both Pro68Ala and Tyr211Ser amino acid substitutions in OXA‐48‐producing strains resulted in decreased susceptibility to CAZ/AVI. Enzyme kinetics showed that, compared with wild‐type OXA‐48, the variant enzyme had >20‐fold higher ability to hydrolyze CAZ, and a >5‐fold reduction in the inhibitory activity of AVI, suggesting that both are responsible for CAZ/AVI resistance. Molecular modeling showed that the hydrogen bond network mediated by water molecules was altered in the variant enzyme. In wild‐type enzymes, Tyr211OH and Thr234O, and Pro235O and Met236O, form hydrogen bonds with central water molecules. In the variant enzyme, however, Ser211 cannot participate in the same H‐bond network because of its less‐space‐filling properties than Tyr211. The loss of H‐bonds could enhance enzyme flexibility, which resulted in increased hydrolysis of CAZ. Moreover, the loss of aromatic stacking resulting from the Tyr211Ser amino acid substitution reduced AVI inhibitory activity (Fröhlich et al., 2019). In addition, OXA‐539, a variant of OXA‐2 with a Asp149 repeat, was found to be resistant to CAZ/AVI (Fraile‐Ribot et al., 2017), as were several *Acinetobacter baumannii* strains carrying class D β‐lactamases, such as OXA‐23, OXA‐40, OXA‐58, and OXA‐66. The inability of AVI to penetrate into their cell membranes prevents AVI from inhibiting these carbapenemases (Yoshizumi et al., 2015).

#### 3.1.5 CAZ/AVI resistance caused by other β‐lactamase variants

In October 2019, an outbreak of CAZ/AVI‐resistant KPC‐2‐producing *K*. *pneumoniae* was reported in Athens, Greece. Resistance of the strain was due to a variant of the Vietnamese extended‐spectrum β‐lactamase‐1 (VEB‐1). Compared with VEB‐1, which had a MIC value for CAZ/AVI of 0.25/4 mg/L, this variant, VEB‐25, with a Lys234Arg amino acid substitution, had a MIC value of 16/4 mg/L. Resistance to CAZ/AVI was due to interference by Arg of the ability of AVI to bind to the active site of the enzyme, thereby attenuating the inhibitory effect of AVI (Galani et al., 2020).

### 3.2 CAZ/AVI resistance associated with changes in bacterial membrane permeability

The bacterial outer membrane proteins OmpF and OmpC, and their homologous proteins, play a role in CAZ/AVI resistance, although these proteins do not constitute the main pathway by which AVI passes through the cell walls of *Enterobacteriaceae* (Pagès et al., 2015).

A study investigating whether mutations in outer membrane proteins affect CAZ/AVI antibacterial activity found that the IS 5 insertion in *ompK36*, or the 134_135 Gly‐Asp insertion in OmpK36, when combined with ESBL such as SHV, TEM, and CTX‐M, are associated with decreased susceptibility of *K*. *pneumoniae* to CAZ/AVI (Shields et al., 2015). The decreased susceptibility of KPC‐producing *K*. *pneumoniae* to CAZ/AVI was not only related to high expression of the *bla*_KPC_ gene, but also to loss of OmpK35, as confirmed by SDS‐PAGE. This indicates that the absence of OmpK35 contributes to the barrier of CAZ to permeate cells (Pagès et al., 2015). The genetic mechanism underlying OmpK35 deficiency varies by Sequence Type. For example, OmpK35 deficiency in ST11 strains may be due to early frameshift mutations and premature termination codons, whereas decreased OmpK35 expression in ST15 strains is associated with negative regulators of *micF* and *ompR*. *MicF* plays an important role in the transcriptional regulation of OmpK35 expression, whereas *ompR* negatively regulates the expression of OmpK35, either directly or through *micF* (Delihas & Forst, 2001).

The increased clinical use of CAZ/AVI has given rise to CAZ/AVI‐resistant strains of *K*. *pneumoniae*. The first CAZ/AVI‐resistant KPC‐3‐producing strain of *K*. *pneumoniae*, KP1244, was compared with the CAZ/AVI‐susceptible strain KP1245. Whole‐gene sequencing showed that both KP1244 and KP1245 have an Arg191Leu substitution in OmpK36, with KP1244 also having a Thr333Asn substitution in OmpK36. A frameshift mutation in *ompK35* results in a truncated, 42‐amino acids OmpK35, resulting in a nonfunctional porin (Humphries & Hemarajata, 2017). Transfer of wild‐type *ompK36* to KP1244 resulted in a 2‐fold decrease in its MIC value for CAZ/AVI, from 32/4 to 16/4 mg/L, whereas transfer of wild‐type *ompK36* to KP1245 did not alter the MIC value. These findings indicate that although Arg191Leu does not affect CAZ/AVI MIC values, the Thr333Asn substitution alters susceptibility to CAZ/AVI. By contrast, transfer of wild‐type *ompK35* to KP1244 and KP1245 reduces their MIC values for CAZ/AVI by 8‐fold, to 4/4 and 0.5 mg/L, respectively. A strain of *Klebsiella oxytoca* with a CAZ/AVI MIC value of 16/4 mg/L lacks the carbapenemase gene, whereas its OmpK36 has several amino acid substitutions (Ala192Pro and Asn229Ser) and deletions (Phe268 and 272_277GlyAspSerAspSerIle deletions; Aitken et al., 2016). Screening of two CAZ/AVI‐resistant strains showed decreased *ompK36* gene expression and a premature stop codon in *ompK35*, resulting in a truncated porin (Castanheira et al., 2017). An alteration in the L3 region of OmpK36 in *K*. *pneumoniae* results in decreased susceptibility to CAZ/AVI (Castanheira et al., 2020), indicating that impaired cell membrane permeability is associated with elevated MIC values to CAZ/AVI. Resistance of *A*. *baumannii* strains to CAZ/AVI is due mainly to the failure of AVI to penetrate the outer membrane (Mushtaq et al., 2010). In addition to OmpC, OmpF, and their homologous proteins, the introduction of a wild‐type Lamb into resistant strains carrying a variant Lamb with Arg33His and Arg374Leu substitutions results in a 2‐fold reduction in the MIC values of these strains for CAZ/AVI, indicating that variant Lamb contributes to resistance to CAZ/AVI (Guo et al., 2021).

### 3.3 CAZ/AVI resistance mechanisms associated with efflux pumps

Overexpression of efflux pumps can reduce intracellular concentration of antibiotics and strain susceptibility to antibiotics (Nikaido & Pagès, 2012). Addition of the efflux pump inhibitor phenylalanine–arginine β‐napthylamide (PaβN) and CAZ/AVI revealed that PaβN did not significantly affect the antibacterial activity of CAZ/AVI, showing that CAZ/AVI enters cells primarily through a passive barrier, with little contribution from active efflux AcrAB‐TolC (Pagès et al., 2015). In contrast to *Enterobacteriaceae*, the presence of PAβN or the efflux pump inhibitor carbonyl cyanide m‐chlorophenylhydrazone (CCCP) reduces the MIC values of some strains of *P*. *aeruginosa* for CAV/AZI, from >32/4 to <0.06/4 mg/L (M. L. Winkler, Papp‐Wallace, Hujer, et al., 2015).

Overexpression of the *mexA*, *mexB*, and *oprM* genes in CAZ/AVI‐resistant strains of *P*. *aeruginosa* isolated from patients with cystic fibrosis altered their MIC values to CAV/AZI from 0.5/4 to 2/4 mg/L. Further analysis showed that the efflux rate of the efflux pump was associated with the MIC value. For example, the MIC values for CAV/AVI of isolates with very slow and faster efflux rates of the efflux pump were 8/4 and 64/4 mg/L, respectively. The mechanism underlying the resistance of these bacteria to CAV/AVI was is due mainly to increased activity of the MexAB–OprM efflux system, resulting from overexpression of *mexA* and overproduction of AmpC (Chalhoub et al., 2018). These findings suggest that efflux pump‐related mutations play a non‐negligible role in the mechanism of CAZ/AVI resistance.

### 3.4 CAZ/AVI resistance mechanisms caused by variants of PBPs

The Leu169Pro amino acid substitution in PBP3 results in a significant increase in the MIC value for CAZ/AVI. Introduction of the wild‐type *ftsl* gene into the resistant strain reduces the MIC value for CAZ/AVI from 128/4 to 4/4 mg/L (Guo et al., 2021). Alterations in the PBP‐encoding gene ftsI are associated with CAZ/AVI resistance (Castanheira et al., 2019). A CAZ/AVI MIC of 8/4 μg/ml was reported against one *E*. *coli* isolate with an unusual TIPY insertion following Tyr344 in penicillin‐binding protein 3 (PBP 3) as the result of gene duplication, which could affect the entry of CAZ (Zhang et al., 2017). In addition, some studies showed that a strain of *K*. *pneumoniae* was continuously cultured in broth medium with CAZ/AVI concentration of 1/4 mg/L under antibiotic selective pressure. The results showed that amino acid substitution of Asp354Ala of mdrA gene encoding PBP2 eventually increased MIC of CAZ/AVI from 0.25/4 to 8/4 mg/L (Karlowsky et al., 2021; Livermore et al., 2018).

## 4 METHODS OF DETECTING CAZ/AVI RESISTANCE

Rapid detection of resistance to CAZ/AVI can be beneficial to the early implementation of appropriate therapeutic interventions. The rapid colorimetric method‐Andrade screening antimicrobial test (ASAT), involving a 3 h incubation with Andrade solution, can rapidly detect bacterial resistance to CAZ/AVI, thereby enabling timely treatment. The ASAT reagent contains NaCl, peptone, and Andrade indicator, with a pH adjusted to 7.4 ± 0.2. Each reaction contains 175 μl of ASAT reagent and a certain concentration of CAZ/AVI, to which is added 25 μl of bacterial suspension, followed by incubation at 37°C for 3 h. Gram‐negative bacteria are detected by fermentation of glucose medium. The presence of a purple‐red color indicates a CAZ/AVI‐resistant strain, whereas a light pink color indicates a CAZ/AVI‐susceptible strain. A selective medium called SuperCAZ/AVI has also been developed to screen for CAZ/AVI‐resistant Gram‐negative bacteria. This assay was based on findings showing that the resistance breakpoints of *Enterobacteriaceae* and *P*. *aeruginosa* to CAZ/AVI are 8/4 mg/L, with the medium optimized to a final CAZ/AVI concentration of 6/4 mg/L. A diluted suspension of bacteria is layered onto culture medium containing CAZ/AVI, and bacterial growth is evaluated after 18 h. SuperCAZ/AVI medium can be used to screen patients for the presence of potentially CAZ/AVI‐resistant strains, allowing the implementation of targeted measures to quickly limit the transmission of infection (Sadek et al., 2020). This culture medium can also be used in epidemiological investigations to assess the prevalence of CAZ/AVI‐resistant Gram‐negative bacteria in populations. The development of new methods of detecting CAZ/AVI resistance may facilitate more rapid detection and treatment of resistant strains. These methods to detect CAZ/AVI resistance are with excellent values of sensitivity and specificity. And these screening methods provide the opportunity to detect CAZ/AVI‐resistant isolates regardless of their resistance mechanisms. They are reliable and economical. However, the specific resistance mechanisms to CAZ/AVI are unclear, so it cannot guide medication concretely.

## 5 CONCLUSION

CAZ/AVI, a combination of β‐lactam and a novel β‐lactamase inhibitor (AVI), has been listed since 2015, which is an important drug used to treat complicated urinary tract infections and complicated intra‐abdominal infections caused by carbapenem‐resistant Gram‐negative bacterial infections. Increases in its clinical use have led to the development of bacterial resistance. According to the global resistance surveillance reports in recent years, the overall resistance rate of Gram‐negative bacteria to CAZ/AVI was low. The resistance rate of *P*. *aeruginosa* to CAZ/AVI was significantly higher than that of *Enterobacteriaceae*. In addition, the resistance rate of CAZ/AVI in carbapenem‐resistant Gram‐negative bacteria was significantly increased. And the resistance rate of CAZ/AVI was significantly different among different continents. By summarizing the mechanisms of resistance to CAZ/AVI, we find that β‐lactamase variants, especially those associated with *bla*_KPC_ gene mutations, are the most widely reported. Other possible mechanisms associated with CAZ/AVI resistance include overexpression of efflux pumps, reduced expression of outer membrane proteins, and gene mutations leading to loss of outer membrane proteins. Finally, we summarized the detection methods of CAZ/AVI resistance.

These studies on the resistance mechanisms to CAZ/AVI will help guide clinical drug use. Currently, Combination drug therapy is mostly used to prevent and treat the emergence of resistant strains to CAZ/AVI in clinical practice, but the treatment lacks specificity. At present, broad‐spectrum metal β‐lactamase inhibitors have been developed for the strains which are natural resistance to CAZ/AVI. As for the treatment of CAZ/AVI non‐naturally resistant strains, research and development of specific medicine may be the trend of future development. Additionally, the development of new methods to detect CAZ/AVI resistance may be beneficial for the treatment and control of drug‐resistant bacterial infections.

## AUTHOR CONTRIBUTIONS

**Luying Xiong:** Writing – original draft (lead); writing – review and editing (lead). **Xueting Wang:** Writing – original draft (supporting). **Yuan Wang:** Conceptualization (lead). **Wei Yu:** Writing – review and editing (supporting). **Yanzi Zhou:** Supervision (lead). **Xiaohui Chi:** Resources (lead). **Tingting Xiao:** Visualization (lead). **Yonghong Xiao:** Data curation (supporting); supervision (supporting).

## FUNDING INFORMATION

This work was supported by the National Key Research and Development Program of China (2021YFC2300300) and the National Nature Science Foundation of China (No. 81971984) and the Key research and development program of Zhejiang Province (No. 2021C03068).

## CONFLICT OF INTEREST

The authors have declared no conflicts of interest for this article.

## RELATED WIREs ARTICLE

Nanoparticle approaches against bacterial infections

## Data Availability

Data sharing is not applicable to this article as no new data were created or analyzed in this study.
